# What is Phenomenological Bioethics? A Critical Appraisal of Its Ends and Means

**DOI:** 10.1093/jmp/jhad001

**Published:** 2023-04-20

**Authors:** Lewis Coyne

**Affiliations:** Library Services, University of Bristol, Bristol, United Kingdom

**Keywords:** *bioethics*, *health and illness*, *Martin Heidegger*, *phenomenology*, *philosophical anthropology*

## Abstract

In recent years the phenomenological approach to bioethics has been rejuvenated and reformulated by, among others, the Swedish philosopher Fredrik Svenaeus. Building on the now-relatively mainstream phenomenological approach to health and illness, Svenaeus has sought to bring phenomenological insights to bear on the bioethical enterprise, with a view to critiquing and refining the “philosophical anthropology” presupposed by the latter. This article offers a critical but sympathetic analysis of Svenaeus’ efforts, focusing on both his conception of the ends of phenomenological bioethics and the predominantly Heideggerian means he employs. Doing so reveals certain problems with both. I argue that the main aim of phenomenological bioethics as set out by Svenaeus needs to be reformulated, and that there are important oversights in his approach to reaching this end. I conclude by arguing that to overcome the latter problem we should draw instead on the works of Max Scheler and Hans Jonas.

## I. INTRODUCTION

Bioethics can be broadly understood as ethical reflection on the practices, policies, and technologies involved in medicine and the life sciences.^1^ Within the discipline the most common approaches track those of mainstream moral and political philosophy—consequentialism, deontology, and liberalism—and tend to strike a balance between four ethical principles that command a general consensus amongst scholars: autonomy, justice, beneficence, and non-maleficence (Beauchamp and Childress, 2019). In the last few years, however, an alternative approach has grown in prominence that draws on quite different philosophical resources, and, in so doing, claims to deepen bioethical reflection. This alternative is *phenomenological bioethics*.

The phenomenological approach to bioethics has existed for as long as the latter discipline itself—the most significant early proponent being the German–Jewish philosopher and founding fellow of the Hastings Center, Hans Jonas (1974, 1985).^2^ In Jonas’ day, however, it was an unorthodox way to conduct bioethical enquiry, and it is only in the last few years that it has increased in visibility. This has largely occurred by building on the now-relatively mainstream phenomenological approach to health and illness. The latter will be discussed at greater length below, but as a preliminary definition, we may say that it explores the patient’s “lived” experience of health and illness, typically with the aim of enriching medical and life scientific practices. As such, the phenomenology of health and illness offers a novel basis for addressing bioethical problems: in contrast to more orthodox approaches to bioethics, which apply the aforementioned set of abstract principles to a given situation, the phenomenological approach begins by attending to the richness and lived quality *of* that situation, hoping to descriptively capture the nuances of its ethical dimensions. Proceeding in this “bottom-up” manner, phenomenological bioethics promises to both complement and productively challenge mainstream, “top-down” bioethics, and therein lies its appeal.^3^

Although there are numerous figures currently working in phenomenological bioethics, the philosopher who has done the most to thematize it from this new vantage point is Fredrik Svenaeus.^4^ In a series of publications Svenaeus (2007, 2010, 2011, 2014, 2018, 2019) has sought to bring phenomenological insights to bear on bioethical issues, with the aim of transcending the limitations of mainstream bioethics. Thus far, however, little critical attention had been paid to either his efforts or those of other phenomenological bioethicists.

Since the phenomenological approach has the potential to enrich bioethical debate, and as Svenaeus’ is the most thoroughly developed recent version of it, the present article will offer a critical analysis of his project—thereby addressing the aforementioned gap in the literature. Because Svenaeus’ version of the approach is fairly novel I will not concern myself here with his fine-grained analyses of specific bioethical issues, but rather the general conceptual means he employs and the overarching end these are intended to serve. This critique is by no means unsympathetic. Nevertheless, I argue that there are problems to be found in both the *terminus a quo* and *terminus ad quem* of his theory: more specifically, that the phenomenological stance he adopts is in certain respects insufficient, and that the aim of his bioethical enterprise is unduly restricted. Neither of these obstacles are insurmountable, however, and so I also make positive suggestions as to how the ends and means of a phenomenological bioethics might be productively reformulated for future research.

## II. THE AIMS OF PHENOMENOLOGICAL BIOETHICS

We shall start with the overarching question of what phenomenological bioethics is supposed to achieve: its *terminus ad quem*. Svenaeus’ answer—the fullest yet offered—is threefold. As is perhaps to be expected, these three aims are derived partly from bioethics (which I assume the reader has a reasonable familiarity with) and partly from phenomenology, which will perhaps require a degree of exposition.

Svenaeus suggests that a phenomenological bioethics firstly provides us with rich descriptions of the “lived experiences of moral conundrums” that arise in clinical situations and life-scientific practices (2018, 9). This aspect follows from the basic orientation of phenomenology itself: a method of philosophical analysis developed primarily by Edmund Husserl (1970), Max Scheler (1973), Martin Heidegger (2010), and Maurice Merleau-Ponty (2012) in the early 20th-century.^5^ While the four figures just mentioned understood phenomenology in diverse ways, engaging in different projects and adopting idiosyncratic vocabularies, a broad definition of the movement may still be provided. Phenomenology attempts to set aside theoretical metaphysical and epistemological prejudices so as to describe phenomena just as they are given to us. This entails neither a turn “inwards” to subjective psychology, nor a turn “outward,” as it were, to a mind-independent reality. Rather, phenomenology examines the *interrelation* of self and world, studying the latter in its manner of appearing to the former. As such, it can be initially understood as a first-person, descriptive enterprise, notably contrasting with the third-person, explanatory perspective of the sciences.

To begin with such an approach can inform bioethics simply by taking the ethical dimension of medical and life-scientific phenomena as its object of study. A phenomenological bioethics of abortion, for example, would focus not on the fetus as an object of scientific study, but rather explore how it appears as a moral patient in its various stages of gestation to the pregnant woman carrying it. Svenaeus claims that by proceeding in this way phenomenological bioethics may, as the first and most basic of its tasks, illuminate how certain core principles of mainstream bioethics—beneficence and justice, for instance—actually appear in and pertain to real-life situations, thereby contributing to our understanding of the salience and lived meaning of those principles.

Phenomenology is not *just* a descriptive enterprise, however—such a conception would amount, as Scheler observed, to mere “picture-book phenomenology” (1973, xix). On the contrary, phenomenology only begins with description, before attempting to systematically account for what is discovered. Thus it can provide us with detailed theories of embodiment, temporality, sociality, and many other issues besides, including—crucially—values, moral norms, and physical and mental suffering. On this basis the phenomenologist may compare what is systematically revealed with more established philosophical theories, potentially exposing the latter’s oversights and shortcomings. In line with this capacity, Svenaeus attributes to phenomenological bioethics a second aim: “to criticize the contemporary set-up of bioethics and offer alternative approaches” (2018, 14).^6^ What might this enterprise entail? For Svenaeus it means that we spell out “the meaning of the good and just in the first place”: accounting for the fullness of these phenomena as they appear in medical and life-scientific situations (2018, 14). On that basis, the phenomenologist can assess whether the standard bioethical principles wholly capture what we have discovered about goodness and justice, or if—as is perfectly possible—further principles and concepts are also required.

The foregoing all follow, as indicated, from the general orientation of phenomenology and its engagement with ethical issues. If, therefore, we accept the method itself as sound, and its extension to questions of practical ethics as legitimate—as, I think, most philosophers would—then none of the above should be *prima facie* objectionable. But what *might* prove contestable is the third aim of the phenomenological approach to bioethics that Svenaeus introduces. Encompassing the first and the second aims, and thereby representing the centerpiece of his theory, Svenaeus suggests that the true goal of phenomenological bioethics is to “scrutinize and thicken the *philosophical anthropology* more or less visibly at work in contemporary bioethics” (2018, 14). But what does this mean, exactly?

“Philosophical anthropology” is a relatively uncommon term in Anglophone philosophy, and Svenaeus does not offer a precise definition of it. This is unfortunate, as it can be understood in at least two different ways (Fischer, 2009). On the one hand, philosophical anthropology understood as a *subject matter* refers to the theoretical study of what human beings essentially are. On the other hand, philosophical anthropology can be understood as a *method* that has its roots in Kant’s thought (2006), and which reached its pinnacle in the first half of the 20th century (Scheler, 2009; Plessner, 2019; Cassirer, 1944; Gehlen, 1988). With admirable ambition, philosophical anthropology in this second sense attempts to synthesize the findings of the humanities and social sciences (*Geisteswissenschaften*) with those of the life sciences (*Naturwissenschaften*) to arrive at the best-informed theory of the human being possible.^7^ Thus the latter is a particular way—but by no means the only one—of pursuing the former kind of philosophical anthropology.

Judging by his remark (to be explained further below) that “[p]hilosophical anthropology is […] a core part of every type of ethical analysis, whether the philosopher acknowledges this to be the case or not,” we may assume that Svenaeus is employing the term in the first sense: referring to a conception of the human being, or, as he also puts it, “a philosophy of personhood” (2018, 16, 18). Since Svenaeus contends that this third purpose of phenomenological bioethics encompasses the first and second, his overall understanding of the approach can be summed up as follows: it is the use of phenomenological description to develop a general conception of the human being “in which the well-known *prima facie* [bioethical] principles can be anchored and critically transformed” (2018, 16, my emphasis).

## III. FOUR POSSIBLE OBJECTIONS TO SVENAEUS’ VERSION OF PHENOMENOLOGICAL BIOETHICS

Svenaeus’ account of phenomenological bioethics seems to fulfill certain desirable criteria. For one thing, it is consistent with the descriptive and critical functions of the phenomenological enterprise. More importantly, perhaps, it opens up an alternative way of conducting bioethical analysis that—although hard to translate into policy—may well be truer to moral problems as they actually arise. However, Svenaeus’ version of phenomenological bioethics is also open to several possible objections, four of which I shall address now. Doing so will add crucial detail to our analysis of his enterprise, and while the first and second objections can, I believe, be fairly easily overcome, the third and fourth require a more substantial adjustment on the part of his theory. All in one way or another concern Svenaeus’ invocation of philosophical anthropology, which is henceforth understood, unless otherwise stated, in the sense of a subject matter rather than a method.

1. The first and most obvious issue to contend with is why phenomenological bioethics should concern itself with philosophical anthropology at all. The short answer is that given above: *every* ethics is already concerned with philosophical anthropology, meaning that Svenaeus’ version of phenomenological bioethics differs only in that it makes explicit what is ordinarily implicit. This is because any branch of philosophy pertaining to human action—ethics, political philosophy, social philosophy—has a general notion of the human being as one of its core presuppositions, and this notion steers it in certain directions without its practitioners necessarily being aware of it (Landmann, 1974; Schacht, 1990). While it would certainly be rash to suggest that all ethicists, or even just all bioethicists, adhere to one shared philosophical anthropology, it is probably fair to say that the *dominant* such content of Anglophone ethics is broadly Lockean and Benthamite: that the human being is a self-conscious individual, in possession of a body, that acts in satisfaction of its preferences. Making this more-or-less implicit theory explicit, exposing any shortcomings, exploring its ethical implications, and offering a fuller alternative based on detailed phenomenological description can only be to the good of bioethics.^8^
2. The second objection is more specific. It runs as follows: Svenaeus claims that a phenomenological description and theory of the good can be subsumed into a new philosophical anthropology—but this is not possible. The reason why is that even if philosophical-anthropological content is a core part or presupposition of ethics, as Svenaeus suggests, ethics is logically distinct from philosophical anthropology. For any ethical theory worthy of the name is not principally concerned with what human beings *are*—the domain of philosophical anthropology—but rather with what they *ought to be and do*. As such, a phenomenological enquiry into the good plainly exceeds the remit of “scrutinizing and thickening” the philosophical anthropology implicit in mainstream bioethics.

The extent to which this objection holds depends, however, on how broad an understanding of philosophical anthropology we adopt. If we take philosophical anthropology to be concerned only with a *formal* understanding of human beings, then a phenomenological bioethics that hopes to scrutinize and thicken the philosophical-anthropological content of mainstream bioethics will only result, at best, in a more accurate formal account. And while this could be bioethically relevant, potentially re-orienting the mainstream theories, it would not itself *have* an ethical dimension, so would fail as an alternative approach. But this is not the way that Svenaeus conceives of philosophical anthropology, nor is there any reason why it should be so conceived. Instead philosophical anthropology is conceived of here as concerned not only with what human beings are in a formal sense, but also with their *worth*. That this is a legitimate position to take is demonstrated by one of the most powerful philosophical anthropologies in human history: the *imago Dei*, which combines both descriptive and normative components. In like fashion—although secular—Svenaeus tells us that the “concept of personhood in [the present] analysis will be connected to an understanding of such concepts as embodiment, vulnerability, *dignity*, and *authenticity*” (2018, 14, emphasis added). Although the first of these features is evidently not normative, and the second only a borderline case, the third and fourth carry significant normative weight. As such, Svenaeus’ approach evades the objection raised, in that to scrutinize and thicken the philosophical-anthropological content of mainstream bioethics’ will be, at least in part, to elaborate on the worth of persons involved in medical and life-scientific situations.

1. The third objection to Svenaeus’ formulation of the phenomenological approach to bioethics concerns the *scope* of philosophical anthropology. The problem, so this line of argument goes, is that Svenaeus equates philosophical anthropology with the study of “the essential components of human personhood,” when the former is in fact concerned with human beings *tout court* (2018, 14). Why might this matter? For the following reason: although human persons are the central objects of moral consideration in bioethical debates, they are not the *only* such objects—far from it. The most obvious counter-examples are human embryos, fetuses, and children or adults lacking core cognitive capacities. None are persons, as that term is typically used by philosophers, but all are human beings, and it is uncontroversial to suggest that any adequate bioethics must encompass them as well.

This objection is anticipated and partly dealt with in Svenaeus’ own work, however. In the concluding chapter of *Phenomenological Bioethics* Svenaeus analyses embryonic and senile forms of human life through the lens of personhood. The connections map out roughly as follows. Embryos, fetuses, and infants are characterized as pre-, very early, and early persons, respectively, while the partially demented, wholly demented, and brain-dead are respectively thought of as late, very late, and post-persons (Svenaeus, 2018, 137–41). I will not go into further detail here beyond noting that what is being progressively gained or lost in each case is the “narrative” sense of self that belongs to personhood (Svenaeus, 2018, 139). Of principal relevance for the issue at hand is that personhood is not conceived of by Svenaeus as an all-or-nothing affair, but rather something that (most) human beings come to be and cease to be, coinciding with our shading into and out of life. What this still excludes, however, are those human beings who will *never* achieve the status of personhood, however long they happen to live for, and whose good must also be considered by bioethicists. Thus, Svenaeus’ equation of philosophical anthropology with the study of the fundamental components of human personhood only partly evades the objection raised.

1. The fourth and final objection is again damaging to Svenaeus’ conception of phenomenological bioethics. Once again, it pertains to his suggestion that the task of phenomenological bioethics is to thicken and scrutinize the philosophical-anthropological content of mainstream bioethics, but this time notes that the beings excluded from this enterprise are non-human organisms.^9^ While human beings are the central concern of bioethics, and quite rightly so, the discipline also deals with animals and even non-sentient forms of life as they feature ethically in medical and life-scientific practices. The most obvious example is the hotly contested issue of using of animals as experimental subjects, but we could also cite the debates surrounding genetic engineering and synthetic biology, the former of which pertains to animals and plants, and the latter of which typically concerns more basic organisms (Coyne, 2020). To be sure, the claim that non-human life has any value beyond the instrumental is a divisive one amongst bioethicists—at least when measured historically—but it would seem consistent with the phenomenological approach that any latent anthropocentricism is interrogated, that our theoretical prejudices be set aside so as to examine the non-human phenomena as they are given to us. Yet if phenomenological bioethics is concerned with philosophical anthropology, and philosophical anthropology understood as the study of human personhood, then the attempt is ruled out in advance. As such, Svenaeus’ formulation of the phenomenological approach to bioethics is limited in a fairly important way.

It seems, then, that Svenaeus’ conflation of philosophical anthropology with the study of personhood is problematic in two respects: it neglects the very possibility of direct ethical consideration of both non-human life and human beings who cannot become persons. How, then, could we reformulate the task of phenomenological bioethics to encompass both groups? Perhaps the most obvious solution is to simply jettison the commitment to scrutinizing and thickening the philosophical-anthropological content of mainstream bioethics. But this might be an overreaction. One alternative—which would admittedly extend Svenaeus’ already lofty ambitions—is to broaden the notion of philosophical anthropology at play, and to do so by taking inspiration from philosophical anthropology understood as a *method*. Many of the great figures of that school of thought set out to define human beings in tandem with an analysis of life as such, differentiating between the latter’s various forms: plant, animal, and human.^10^ In so doing, they were able to develop an understanding of both human uniqueness (of which personhood is typically seen as a part) *and* the extent to which being human overlaps with other forms of life. Similarly, if we were to phenomenologically pursue a philosophical anthropology (as a subject matter) with reference to a philosophical biology, then we would solve the problem with Svenaeus’ account identified above: phenomenological means could be used to scrutinize and thicken the philosophical-anthropological content of contemporary bioethics, as Svenaeus suggests, but since the philosophical anthropology in question would be connected to a philosophy of *life*, this would truly amount to a phenomenological *bio*-ethics. Since, moreover, this philosophical anthropology would be concerned with understanding human beings and non-human life *normatively*, it would also be a truly phenomenological bio-*ethics*. In this way, then, the *terminus ad quem* of Svenaeus’ phenomenological bioethics may be reformulated to overcome the limitations of its original expression, and thereby stand a better chance of fully living up to its promise.

## IV. THE PHENOMENOLOGY OF HEALTH AND ILLNESS

We shall now turn to the *terminus a quo* of Svenaeus’ phenomenological bioethics: his choice of phenomenological means to the end of a thickened philosophical anthropology for bioethics. As indicated, Svenaeus works from a phenomenology of health and illness—or suffering—and so we must firstly look at what the latter itself entails before drawing any philosophical-anthropological conclusions from it.

As Svenaeus himself notes, phenomenological insights into health and illness can be found in the works of the method’s earliest and greatest proponents: Husserl, Scheler, Heidegger, Sartre, Merleau-Ponty, and so on (2018, 3). However, it was only much later that figures such as Drew Leder (1990) and S. Kay Toombs (1992) fully thematized and developed the phenomenology of health and illness, and only later again that it attained a relatively mainstream status. Regardless, its aim throughout has been to explore the phenomena of physical and mental health and illness from a first-person perspective, reflecting on the lived body and the world in which it is entangled to that end.

In the last decade or so, as Svenaeus again correctly observes, two broad strands have emerged that focus on different figures from the phenomenological tradition: one that broadly follows Merleau-Ponty and another that draws heavily on Heidegger. Svenaeus holds that the difference between the two strands is primarily one of “emphasis and terminology” regarding the focal point of a phenomenology of health and illness (2019, 462). More specifically, he notes that the first, Merleau-Ponty-inspired group, tends to define illness as a rupture of the lived body’s intentional acts and habits, while the second, Heideggerian camp—to which Svenaeus (2000) himself belongs—conceives of illness as an uncanny manner of being-in-the-world. Since these are highly technical terms their respective meanings will be brought out through a concrete example, drawn from my own experience: suffering from a depressive episode.

Although depression is typically conceived of—both by medical professionals and the general public—as a mental illness, the Merleau-Pontian would stress that it is also a *bodily* illness to its core (Carel, 2016). More specifically, it is an illness of the lived body: that is, the body as we actually live through it, rather than the body as it appears to scientific representation. Such a description might take the following form. A depression partly manifests itself as—indeed, partly *is*—an inability to comport oneself in the usual, taken-for-granted fashion. Routine tasks such as getting out of bed, washing, shaving, or making a meal, are all obstructed from within by a deep lacklustreness. Associating with others in an everyday fashion, such as chatting face-to-face, equally feels motivationally impossible. Just imagining doing any of these takes such effort that the attempt often fails to get underway. From this new vantage point it then becomes clear that a part of being in good spirits was the effortlessness of action, with the largely inconspicuous body allowing for relations with other people and the free engagement with things. In the latter state one’s bodily being has a certain *lightness* and ease about it that is only truly seen—and appreciated—when it gives way in depression to a dull heaviness.

The Heideggerian may well agree with the above, but with the phenomenological resources provided by the master would identify the locus of depression elsewhere: namely, the peculiar way in which the world is disclosed to us (Ratcliffe, 2015). As Heidegger’s thought is so dependent on neologisms, etymological wordplay, and otherwise obscure terminology, I shall attempt to set out the basic parameters of his thought and how it allows for a phenomenology of health and illness.

Heidegger’s lifelong concern was the question of the meaning (*Sinn*) of being, and in his early, phenomenological work he sought to lay the groundwork for an answer to it with a “fundamental ontology” (2010, 35). The latter referred to an analysis of the being of *Dasein*: the entity that is uniquely capable of posing the question of the meaning of being. In his masterpiece, *Being and Time*, Heidegger described the being of *Dasein* as “being-in-the-world” (2010, 57). With this formulation he hoped to capture the fact that *Dasein* is not an ego or subject distinct from a world of objects, but that it *is* only *as* engagement with entities belonging to the world. Part of *Dasein*’s being-in-the-world is its “attunement [*Befindlichkeit*]”, which meant, for Heidegger, *Dasein*’s finding himself in the world in a particular way (2010, 130). Attunement has a more conspicuous and readily comprehensible counterpart in being claimed by a mood. According to Heidegger’s phenomenological analysis moods are not to be understood as subjective states, but instead as “a fundamental manner and fundamental way of being, indeed of being-there [*Da-sein*]” (1995, 67). One example given is boredom. “Profound boredom,” he notes, “drifting here and there in the abysses [*Abgründen*] of our existence like a muffling fog, removes all things and men and oneself along with it into a remarkable indifference. This boredom reveals beings as a whole,” he claims, because all things show up through it, are colored by it (Heidegger, 1977, 101).

In profound boredom, we have a mood comparable to depression. Like boredom, depression reveals beings as a whole to us, but it does so in its own peculiar way. In a depression the environing world shrinks back from its everyday immediacy and becomes uncanny; it is there for us but somehow distant and intangible, almost out of reach. Fundamental to this shift is that once all-consuming tasks and projects retreat from view. Things may still be there, but the appeal they make is dimmed and frequently goes unheeded; people are there, too, but typically as an oppressive intrusion on our solitude. Once again, from this perspective we are able to truly see and appreciate how beings formerly showed up as vivid and captivating, even enthralling; now, they are only mute and inconsequential.

The foregoing sketches will hopefully suffice as an indication of the two central approaches to the phenomenology of health and illness. As indicated, Svenaeus opts for the latter, Heideggerian variety as his principal means to developing a thicker philosophical anthropology for bioethics in both formal and normative respects. The formal philosophical-anthropological content that Svenaeus develops with the aid of Heidegger’s thought focuses on the various aspects of *Dasein*’s being-in-the world. Thus he stresses our being-in-the-world with others, our temporally structured being-toward-death, and our “being-in-the-body”, since these are all levels at which health, illness, and suffering manifest (2018, 35). This is supplemented with insights from Charles Taylor on our narratively structured sense of self (ch. 2), Jean-Paul Sartre on being ill (ch. 3), and Hans-Georg Gadamer on medical encounters (ch. 4). In addition to this already broad array of sources, Svenaeus draws on medical research into pathologies of the mind and body (ch. 3) as well as a biologically-informed account of human ontogenesis (ch. 6). On the normative side, Svenaeus largely builds on Heidegger’s concern that human beings may be objectified and instrumentalised by the medicalization of core aspects of our being, reinforcing the latter with Aristotle’s understanding of *phrónēsis* and Edith Stein’s account of empathy (ch. 4). In this way he hopes to elucidate fundamental aspects of personhood, and thereby develop a philosophical anthropology that is truer to the richness of human life than the Lockean–Benthamite model currently predominant in Anglophone bioethics.

Since my focus here is on the general architecture rather than the detail of Svenaeus’ theory, I will not go into greater depth at this point other than to stress that, to a degree, Svenaeus’ choice of phenomenological means allows him to fulfill the promise of his phenomenological bioethics. But he is not *entirely* able to do so, and this is due to the decision to place Heidegger at the center of his approach. As stated, Svenaeus suggests that the difference between the Merleau-Pontian and Heideggerian phenomenologies of health and illness is principally one of emphasis and terminology. This is debatable, however. On the contrary, there is good reason to think that there are structural disadvantages to opting for a principally Heideggerian phenomenology of health and illness—disadvantages that emerge most clearly when this phenomenology is applied to the bioethical domain, and with the specific aim of scrutinizing and thickening the philosophical-anthropological content of mainstream bioethics.

The basic problem afflicting Svenaeus’ approach is that Heidegger never adequately accounted for ethics or for our corporeal bodies; indeed, he never sought to. In a lecture course contemporaneous with the publication of *Being and Time*, Heidegger stressed that fundamental ontology does not prepare the ground for the “proper philosophical disciplines of logic, ethics, aesthetics, and philosophy of religion,” but rather contains within it “the possibility of reversing the alienation of philosophy into these disciplines,” in that each is “called into question and eliminated precisely by phenomenology itself” (1982, 3). Yet more straightforwardly, in the following year’s lecture course he stated that his “analysis proceeds solely with the purpose of a fundamental ontology […]. The issue is therefore neither one of anthropology nor of ethics” (1984, 136).^11^ As such, Svenaeus’ reliance on Heidegger’s thought leads to philosophical-anthropological difficulties in both formal and normative respects. Let us look at these aspects in turn.

## V. HEIDEGGER ON EMBODIMENT

As stated, in Heidegger’s early thought the body barely featured at all. *Being and Time* contained only passing references to embodiment, and the purpose of these remarks was only to emphasize the way in which *Dasein* is *not* corporeal: namely, the Cartesian sense of an “I-thing encumbered with a body” (2010, 104). By the time of its sequel, *Kant and the Problem of Metaphysics*, Heidegger had arrived at a more positive proposal insofar as he admitted that the “human being […] to a certain extent has been fettered in a body” (1997, 203). But exactly what this fetteredness consisted of, and why it was framed in such a Cartesian manner, went unexplained. It was only in Heidegger’s later work that embodiment was discussed in greater detail, and even then at only two points in his voluminous writings: the lectures on Nietzsche given from 1936 onward (1979/1984, 1987/1982), and the *Zollikon Seminars* of 1959–69 (2001). Given that the latter text is concerned with medicine and modern science Svenaeus quite understandably places a great interpretative weight on it, and so we shall look in more detail at what Heidegger says there.

Whereas in *Being and Time* Heidegger accounted for *Dasein*’s being-in-the-world in acorporeal terms, in the *Zollikon Seminars* we are told that “we must characterize all comportment of the human being as being-in-the-world, determined by the *bodying forth of the body*” (2001, 90–1, my emphasis). What did he mean by this latter turn of phrase? Like Merleau-Ponty, Heidegger there draws a firm distinction between the corporeal body (*Körper*) and the lived body (*Leib*), that is, between the body as objectified—including by the natural sciences—and the body that we actually live as. This insight was by no means original: both Husserl and Scheler had already phenomenologically described it, and it was arguably present even in Schopenhauer’s *The World as Will and Representation* (1958). Nevertheless, its belated and brief—though by no means superficial—appearance in Heidegger’s work does at least indicate how the fundamental ontology of *Being and Time* may be rethought on those lines.

Heidegger observes that the *Leib* does not perfectly coincide with the *Körper*, which ends with the surface of the skin, but rather proceeds beyond it by “bodying forth [*Leiben*],” as he puts it (2001, 97). All of the components of our being-in-the-world—attunement, being-with, being-toward-death, and so on—are shaped by this bodying forth of the body (*Leib*), which is immediately given to us and only open to description from a first-person perspective. On this basis, as Svenaeus argues, a Heideggerian phenomenology of health and illness may ground its insights in the appearance of our lived body, stressing the ways in which health and illness manifest in alterations to our bodying forth in the world.

Now, the main problem with Heidegger’s later thinking of the body is not its brevity, but rather its denial of any connection, beyond the coincidental, between the *Leib* and the *Körper*. Although Heidegger indicates that being-in-the-world is determined by the bodying forth of the lived body, he is adamant that bodying forth itself is *not* determined by the corporeal body, as one might expect. Rather, bodying-forth is governed by “the horizon of being within which I sojourn”—that is, *Dasein*’s existence (Heidegger, 2001, 87). It should be stressed that “existence” (*Existenz*) is a Heideggerian *term d’art*, referring not to *Dasein*’s objective presence but rather the fact that its own being is an issue for it, which is inseparable from *Dasein*’s largely tacit grasp of the “ontological difference” between being (*Sein*) and beings (*Seiendes*) (Heidegger, 1982, 319). Moreover, because Heidegger holds that this openness to being “cannot be derived from anything else,” he is led to the conclusion that *Dasein*’s existence is the condition of possibility for its bodiliness, rather than the other way around (2001, 233). This is stated most explicitly in the following passage:

Therefore, regarding the whole bodiliness, we must repeat what we have mentioned before about seeing and our bodily eyes: We are not able to “see” because we have eyes; rather we can only have eyes because, according to our basic nature, we are beings who can see. Thus, we would not be bodily [*leiblich*] in the way we are unless our being-in-the-world always already fundamentally consisted of a receptive/perceptive relatedness to something which addresses us from out of the openness of our world, from out of that openness *as which we exist*. […] It is only because of our *Dasein*’s essential direction [*Ausrichtung*] that we are able to distinguish between in front of and behind, above and below, left and right. It is due to the same directedness [*Ausgerichtetsein*] toward something addressing us *that we can have a body at all*, better: To *be of a bodily nature*. We are not first of a bodily nature and then from it have what is in front and behind, and so forth. (Heidegger, 2001, 232, my emphasis)

In addition to any metaphysical objections we might have with this understanding of embodiment, it poses a problem for Svenaeus’ enterprise—namely, that it allows for no foundational connection between our lived body and our corporeal body, our being-in-the-world and our organismic being. Why is this an issue? Because it means that Heidegger’s analysis overlooks the extent to which our being-in-the-world may be illuminated by the life sciences, courtesy of what the latter can tell us about the *Körper*. Judging by his recourse to medical and biological findings, Svenaeus himself holds that the fact that our lived body largely (though by no means entirely) coincides with the corporeal body cannot be a *mere* coincidence, and, moreover, that it is essential to an adequate philosophical anthropology that this connection is explored and drawn out. I wholly agree—but a Heideggerian analysis of the body is theoretically at odds with the attempt. Whatever truth Heidegger’s understanding of the body might contain, Svenaeus’ reliance on it thereby undermines his attempt to develop a thicker philosophical anthropology for bioethics in the formal sense.

## VI. HEIDEGGER ON ETHICS

The second major problem with using Heidegger’s phenomenology to develop a deepened philosophical anthropology for bioethics concerns *ethics*. We noted above that there is no reason why a philosophical anthropology cannot regard human beings as bearers of certain kinds of values, revealed by phenomenological analyses of how these show up in medical and life-scientific encounters. As such, a philosophical anthropology can be inherently normative, and therefore of direct relevance to bioethics, provided that the phenomenological means employed to develop it are capable of exploring this dimension. Unfortunately, however, Heidegger’s philosophy is in this respect inadequate, for the following reasons.

As indicated, Heidegger regarded his early phenomenological project not as laying the groundwork for a possible ethics, but rather as dissolving the latter through its assimilation into fundamental ontology. Since fundamental ontology is concerned with explicating the question of the meaning of being, it has nothing substantial to say about the good, the just, and the right, and it is for this reason that the various attempts to derive an ethics from Heidegger’s early work are so unconvincing.^12^ The trio of concepts from *Being and Time* that are most commonly invoked as the collective basis for an ethics are care, authenticity, and the call of conscience. To be sure, each term has strongly normative overtones, and Heidegger’s analysis of the first certainly makes logical room for the possibility of ethical conduct. But all are systematically “ontologized” and made neutral with regards to any inherent normative content.^13^ “Care,” for example, is merely the name for *Dasein*’s orientation toward its own “potentiality-of-being,” and accordingly allows for both ethical and unethical conduct (Heidegger, 2010, 227). Likewise, according to Heidegger, the call of conscience “discloses nothing that could be positive or negative as something to be taken care of, because it has to do with an ontologically completely different being, namely, *existence*” (2010, 282). Of right and wrong, virtue and vice, justice and injustice, the early Heidegger had nothing to say.

When Heidegger eventually spoke of ethics at some length, in the “Letter on Humanism,” it continued in this ontologising vein.^14^ There he suggested that ethics properly understood—as the Greek *ēthos*—bears little resemblance to what goes by that name today. Far from referring to utility, duties, or even moral character, *ēthos* supposedly means *Dasein*’s “abode,” which “contains and preserves the advent of what belongs to man in his essence” (Heidegger, 1977, 233). But what is it that essentially belongs to the human being? Heidegger’s answer is existence (*Existenz*): our unique openness to being. Thus, the *ēthos*, according to Heidegger, points away from any concern with the good life or what we owe to others, and leads instead to the ethically neutral terrain of ontology—hence his claim that humanity is the “shepherd of Being,” whose task is to watch over the openness to being that is its prerogative (1977, 210).

Since Heidegger’s ethical reflections are so meager, Svenaeus quite understandably seeks to depart from them in two ways. The first, already mentioned above, is via Edith Stein’s phenomenology of empathy, which Svenaeus uses to illuminate the ethical dimension of the medical encounter. Thus Svenaeus argues that empathy is “not merely a kind of ethical icing on the cake,” but rather “one of the basic capacities that makes the doctor able to understand what the reasons for complaints and suffering are about and what can and ought to be done to help the patient in the best possible way” (2018, 61). However, the problem with this move, which Svenaeus himself acknowledges, is that empathy is *not obviously ethical at all* (2018, 59). This judgment, which is admittedly very much at odds with public sentiment, is based on the observation that since empathy is to do with “an imaginative reconstruction of another person’s experience” it is in fact an *epistemic* capacity (Nussbaum, 2001, 301–2). That is to say: empathy entails that one understands what another is feeling, but not that one necessarily *cares about* what the other is feeling (Goldie, 2000; Stocker, 1996). For empathy to extend to morality a further, logically distinct emotion is needed, such as concern, sympathy, or solidarity, all of which entail that the other person’s plight actually *matters* to us. For this reason, empathy would seem to be at best necessary for ethics, but not sufficient.

To overcome this problem, Svenaeus departs from Heidegger’s ethics in a second way: namely, by giving a normative twist to the latter’s philosophy of technology. This move is more successful, thanks to supplementary insights drawn from Hans-Georg Gadamer’s associated concerns with technological instrumentalism in the healthcare professions. Certainly, Heidegger’s own philosophy of technology probed more deeply than any before or since into the essence of technology, which he defined as the “enframing” or “setting-upon” of beings to extract a pre-determined outcome from them (1977, 301, 296). However, for Heidegger the pressing issue was how the essence of technology has come to define our relation to being, *not* how the enframing of different kinds of beings does varying degrees of violence to them. This oversight emerged most damagingly with his notorious 1949 declaration that “[a]griculture is now a mechanised food industry, in essence the same as the manufacturing of corpses in gas chambers and extermination camps” (quoted in Rockmore 1992, 241). Because his philosophical concern was always ontological—the question of being—overtly ethical issues were foreclosed to him. Gadamer does go one step further, however, expressing concern for “the unique value of the individual” (1996, 81). In an ethical twist on Heidegger’s philosophy of technology, he states that a “loss of personhood […] happens within medical science when the individual patient is objectified in terms of a mere multiplicity of data” (Gadamer, 1996, 81). This welcome reference to the worth of the person allows Svenaeus’ use of empathy to extend to ethics: we may now say that the medic ought to empathize with the patient, and circumvent an instrumental enframing, in order to respect rather than obscure the unique value of their personhood.

The above is an attractive formulation. But, unfortunately, it is once again at odds with Heidegger’s thought, as the latter regarded the very idea of empathy as resting on an “obviously incorrect concept”—namely, the “pure fabrication” that is the Cartesian ego, between two or more of which the empathic encounter is supposed to take place (2001, 111). For Heidegger, as we have seen, the fallacious modern idea of the subject was to be undercut by his analysis of *Dasein*’s being-in-the-world, which had being-with as one of its core components. Thus, Heidegger’s analysis of *Dasein* cannot incorporate the notion of empathy in the way that Svenaeus would like, and the attempt to give Heidegger’s thought an ethical dimension through it fails at the outset.

## VII. CONCLUSION: PHENOMENOLOGICAL BIOETHICS BEYOND HEIDEGGER

Nothing argued in the previous two sections is meant to imply that a phenomenological bioethics cannot, or ought not, draw on Heidegger’s work. On the contrary: Svenaeus demonstrates that aspects of Heidegger’s analysis of being-in-the-world can generate a plausible phenomenology of health and illness, and that the latter’s philosophy of technology is of direct relevance to bioethics. But any phenomenological bioethics that broadly takes Heidegger’s work as its *terminus a quo* will require significant supplementary insights in order to compensate for his inadequate treatment of ethics and our embodiment—insights that will also have to be compatible with Heidegger’s unique enterprise. While many of Svenaeus’ additional theoretical resources are of this kind, neither scientific findings regarding the body as *Körper* nor the phenomenology of empathy count as such.

So as to conclude in a more constructive spirit, I would like to point toward how the reformulated purpose of phenomenological bioethics, developed in Section 3, might be arrived at. There it was suggested that phenomenological means could be used to thicken the philosophical anthropology employed by mainstream bioethicists in two respects: formally, in terms of the constitution of the human being as distinguished from non-human forms of life, and normatively, in terms of the dignity or worth of the person (again, as distinguished from non-human life). My proposal is that we develop a new *terminus a quo* for this reformulated *terminus ad quem* with the help of two philosophers, both of whom have already been mentioned: Max Scheler and Hans Jonas. I shall briefly set out why these figures might prove to be more fruitful sources of inspiration than Heidegger.

Firstly, there is the issue of embodiment. While Merleau-Ponty (1962, 2012) is, with good reason, the best-known phenomenologist on this topic, comparable ideas may nevertheless be found in Scheler and Jonas’ work. Unlike Heidegger, both thinkers phenomenologically investigated not only the lived body’s role in our being-in-the-world, but its connection to the corporeal body also.^15^ A generation before Merleau-Ponty, Scheler explored the lived body–corporeal body dichotomy in some detail, and, crucially, sought to trace points of connection and difference between the human form of life and its non-human antecedents (1973, 2008a, 2009). In Jonas’ (1966, 2016) work, too, we find a phenomenological investigation into the biological foundations of human existence, which, much like Scheler’s, traces the development of the human form of life through vegetative and animal being. Were this connection between the *Leib* and the *Körper* to be adequately established, we might—at least in principle—be able to connect phenomenological insight to the realm of medical and life-scientific intervention, as would seem desirable for a phenomenological bioethics. That this would not entail any commitment to methodological naturalism, which Heidegger rightly abhorred, is amply demonstrated by Jonas and Scheler’s work, not to mention the comparable thought of Merleau-Ponty and Plessner.

Secondly, there is the crucial issue of ethics. Clearly, any phenomenological bioethics ought to contain an account of the worth of both human and non-human forms of life, how this worth appears to us in the presence of the other, and the ways in which it can be respected or violated. Once again, we find such descriptions in the works of Scheler and Jonas. The former phenomenologically describes the manifold kinds of value, their bearers, and their hierarchical appearance, which runs from the values of things, on through to the values of life as such, and culminates in the value of the person: the non-objectifiable center of intentional acts grounded in the lived body. Jonas (1984), meanwhile, undertakes a complementary analysis of the dignity of human as well as non-human life, the former of which derives from our capacity for morality and is revealed through the phenomenon of responsibility for the other. In his pioneering essays on bioethics, moreover, Jonas (1974, 1985) describes how certain medical and life scientific practices run the risk of objectifying and instrumentalizing human beings, thereby allowing for a direct application to contemporary bioethical debates.

Scheler, who is in any case due a revival, and Jonas, who has never received the attention he deserves from Anglophone philosophers, would thereby seem to be ideal sources of inspiration for a new phenomenologically-derived philosophical anthropology for bioethics.^16^ We might still draw on Heidegger, where doing so is compatible with their thought, but this different *terminus a quo* would allow us to better flesh out both formal and normative aspects of human and non-human life. In this way we might offer richer descriptions of ethical problems as they appear in medical and life-scientific situations, expose the philosophical-anthropological inadequacies of mainstream bioethics, and build a more comprehensive account of human (and non-human) beings by which to guide medical and life-scientific practice—thereby living up to the radical promise of phenomenological bioethics as indicated by Svenaeus.

## Footnotes

1 Medicine is here distinguished from the life sciences for the reason given by Hans-Georg Gadamer in *The Enigma of Health*: namely, that the former is “a peculiar kind of practical science for which modern thought no longer possesses an adequate concept” (1996, 39).

2 For accounts of Jonas’ bioethics see Hauskeller (2015) and Coyne (2021, ch. 7). Jonas’ doctoral student Richard Zaner (1988) is perhaps the most notable phenomenological bioethicist belonging to the subsequent generation.

3 Phenomenological bioethics can be understood, therefore, as one possible way of critiquing what has been called bioethical “principlism” (Clouser and Gert, 1990).

4 In addition to Svenaeus, we might think of Rehman-Sutter, Porz, and Scully (2012), as well as a number of the contributors to Meacham (2015) and Ferrarello (2021).

5 I freely admit that readers familiar with phenomenology might find Scheler’s inclusion on this list surprising. However, Scheler was central to its initial development and opened up pathways of investigation that both Husserl and Heidegger overlooked—facts that, to my mind, go somewhat unacknowledged today. Moran (2000), for instance, excludes Scheler almost entirely from his lengthy textbook on the tradition.

6 Of course, other approaches to bioethics can serve the same critical function. For example, Leon Kass—another of Jonas’ students—sought to do precisely this with his *Life, Liberty and the Defense of Dignity*, and its call for a “richer ethic of *bios* tied to a richer *logos* of *bios*” (2002, 21).

7 This, at least, is my attempt at a concise definition of philosophical anthropology. Scheler, who spearheaded its revival in the 1920s, describes it as “a basic science which investigates the *essence* and *essential constitution* of man, his relationship to the realms of nature (organic, plant, and animal life) as well as to the source of all things, man’s metaphysical origin as well as his physical, psychic and spiritual origins in the world, the forces and powers which move man and which he moves, the fundamental trends and laws of his biological, psychic, cultural, and social evolution, along with their essential capabilities and realities” (1958, 65).

8 For a comparable hermeneutic-phenomenological argument regarding the role of biology in bioethics, see Griffiths (2021).

9 This is with the possible exception of any *animal persons*, such as mature adult members of the great ape species. If such beings are indeed persons, then Svenaeus’ equation of the philosophy of personhood with philosophical anthropology would entail that the latter also encompasses some non-human beings—which renders the very term “philosophical *anthropology*” nonsensical. I thank Jack Griffiths for bringing this point to my attention.

10 Indeed, their search for the *differentia specifica* of human beings drew heavily on the work of a group of biologists and zoologists with whom their work overlapped, most notably Jakob von Uexküll (2010) and Adolf Portmann (1990).

11 With varying degrees of force, Heidegger repeatedly distanced his own project from that of philosophical anthropology. See also Heidegger (1995, 157, 1997, 147–9, 2010, 17).

12 For example, Guignon (1993); Hodge (1995); Olafson (1998); Hatab (2000).

13 Perhaps the most egregious example of this tendency is Heidegger’s reading of Aristotle’s *Nicomachean Ethics* in the *Basic Concepts of Aristotelian Philosophy* (2009). Gonzalez (2006) gives an excellent account of how Heidegger’s creative etymological exercises strip Aristotle’s thought of any normative content.

14 Earlier indications of his position could be found in *The Fundamental Concepts of Metaphysics* (1995), and *Introduction to Metaphysics* (2000).

15 The one obvious exception to this statement is Heidegger’s fascinating interpretation of animal environments (*Umwelten*) in *The Fundamental Concepts of Metaphysics* (1995). Even here, however, Heidegger would think only of animals in terms of corporeality, and not *Dasein* also.

16 For example, Scheler’s (2008b) analysis of sympathy, which he takes to be based on our fundamental and primordial membership of an interpersonal world, would be a welcome counterweight to the current (and in my opinion, largely misplaced) focus on empathy in Anglophone phenomenology.
